# Test-retest reliability of knee extensor rate of velocity and power development in older adults using the isotonic mode on a Biodex System 3 dynamometer

**DOI:** 10.1371/journal.pone.0196838

**Published:** 2018-05-03

**Authors:** Stijn Van Driessche, Evelien Van Roie, Benedicte Vanwanseele, Christophe Delecluse

**Affiliations:** 1 Department of Kinesiology, Physical Activity, Sports and Health Research Group, KU Leuven, Leuven, Belgium; 2 Department of Kinesiology, Human Movement Biomechanics Research Group, KU Leuven, Leuven, Belgium; University of L’Aquila, ITALY

## Abstract

Isotonic testing and measures of rapid power production are emerging as functionally relevant test methods for detection of muscle aging. Our objective was to assess reliability of rapid velocity and power measures in older adults using the isotonic mode of an isokinetic dynamometer. Sixty-three participants (aged 65 to 82 years) underwent a test-retest protocol with one week time interval. Isotonic knee extension tests were performed at four different loads: 0%, 25%, 50% and 75% of maximal isometric strength. Peak velocity (pV) and power (pP) were determined as the highest values of the velocity and power curve. Rate of velocity (RVD) and power development (RPD) were calculated as the linear slopes of the velocity- and power-time curve. Relative and absolute measures of test-retest reliability were analyzed using intraclass correlation coefficients (ICC), standard error of measurement (SEM) and Bland-Altman analyses. Overall, reliability was high for pV, pP, RVD and RPD at 0%, 25% and 50% load (ICC: .85 - .98, SEM: 3% - 10%). A trend for increased reliability at lower loads seemed apparent. The tests at 75% load led to range of motion failure and should be avoided. In addition, results demonstrated that caution is advised when interpreting early phase results (first 50ms). To conclude, our results support the use of the isotonic mode of an isokinetic dynamometer for testing rapid power and velocity characteristics in older adults, which is of high clinical relevance given that these muscle characteristics are emerging as the primary outcomes for preventive and rehabilitative interventions in aging research.

## Introduction

In geriatrics and aging research, measures of muscle function are commonly used to screen for an age-related loss of muscle strength and power and to evaluate the effectiveness of exercise interventions [1, 2]. Hence, measures of muscle function should be consistent and free from error. This is especially relevant for the knee extensor muscles in older adults, as they are crucial in a number of functional and locomotor tasks such as walking, stair climbing, chair rising, balance control and fall prevention [3–5].

To date, the gold standard for measuring muscle function in clinical practice and research settings is isokinetic dynamometry [6, 7]. Isokinetic testing allows maximal strength production at a constant predetermined velocity throughout a selected joint’s range of motion. It has been shown that maximal strength is an important variable to be tested for monitoring loss of muscle strength in the elderly [8]. Previous studies have shown highly reliable test results for knee extension strength measurements using the isokinetic mode [9–14]. In addition to the isokinetic mode, an isokinetic dynamometer can operate in an isometric and an isotonic mode [1, 15]. In the former, maximal strength is produced in a predetermined position without limb movement. In the isotonic mode, muscle contractions are performed at a predetermined resistance and a variable velocity. Considering that most daily activities do not include a constant predetermined velocity or a fixed position as in isokinetic or isometric tests, isotonic tests may better reflect daily activities that involve moving a specific object (weight) and include an acceleration component. The isotonic mode allows for single, explosive movements and can be considered a safe and objective measure of velocity-dependent muscle power [15]. Moreover, isotonic tests can be performed at a certain percentage of the isometric maximum, allowing for power production across the entire power-load relationship. These tests can be considered as relative tests of a subject’s ability (i.e. maximal isometric strength) to generate power. In addition, isotonic tests can also be conducted in an unloaded condition. These unloaded tests, interpreted as absolute tests because every subject has to push the same absolute load, are shown to be a key component in the onset of functional difficulties in the elderly [16]. The ability to address relative and absolute power production in one test mode is unique. Although isotonic training and testing has been performed on common weight stack machines for years, little is known about the isotonic mode of an isokinetic dynamometer, which allows for better standardization, accurate continuous measurement of torque and velocity in a predetermined range of motion and reduced muscle soreness (i.e. concentric only) [17]. The growing amount of studies investigating muscle function of the ankle plantar- and dorsiflexors support the idea of using the isotonic mode on an isokinetic dynamometer for testing muscle function [15, 17, 18]. However, little is known about the reliability of isotonic measures of knee extensor function, in particular in older adults.

Irrespective of test mode, current research agrees that muscle power, in particular its velocity component, is a better predictor of functional performance among older adults than maximal strength [16, 19]. In addition, early age-related changes appear to be more pronounced in power and velocity production than in maximal force generating capacities, resulting in an age-related slowing of muscles [20, 21]. Fundamentally, the majority of these studies investigating muscle function in old age express their results in terms of maximal values of strength, velocity or power. However, recent insights seem to suggest that rapid force production is more functionally relevant [22, 23]. Parameters that reflect the ability to produce force rapidly correspond to the short response times (i.e., less than ~200ms) that are normally available to accelerate the limbs during many functional tasks [24]. Although it has recently been shown that time-dependent measures of isotonic muscle function decline more during aging than maximal output measures [25], there is, to our knowledge, virtually no research that focused on the reliability of these measures.

Therefore, the present study aimed at determining relative and absolute test-retest reliability of knee extensor muscle function using the isotonic test mode on a Biodex System 3 dynamometer in older adults (65–82 years). A particular novelty of this study is the focus on time-dependent measures of muscle function (i.e. RVD and RPD) and their evaluation across loads.

## Materials and methods

### Participants

According to the recommendations of Hopkins et al. [26] on sample size for reliability studies, we aimed at recruiting at least 50 participants through advertisements. Participants were community-dwelling older adults aged between 65 and 85 years old. Exclusion criteria were pathologies that prohibit a maximal strength test, such as severe cardiovascular disease, artificial hip or knee, acute hernia, infection or tumor (diagnosed by the subjects’ general practitioner). Sixty-three older adults (♂: n = 27, age = 73 ± 4 years, body mass = 83 ± 10 kg; ♀: n = 36, age = 72 ± 3 years, body mass = 69 ± 8 kg) volunteered to participate in the study. All subjects gave written informed consent. The study was approved by the University’s Human Ethics Committee (“Commissie Medische Ethiek van de UZ KU Leuven S52221”) in accordance with the declaration of Helsinki.

### Design

A repeated measures protocol was designed to assess test-retest reliability of the isotonic mode at 0%, 25% and 50% of maximal isometric strength on a Biodex System 3 dynamometer evaluating knee extensor peak velocity (pV), peak power (pP), rate of velocity (RVD) and power (RPD) development [27]. The test and retest session were conducted in the same lab on two separate occasions. The time interval between test and retest was 1 week (same time of day and same weekday). All measurements were performed by the same experienced rater.

### Procedures

#### Dynamometry

Measurements of isometric knee extension strength as well as velocity capacities of the knee extensors across different loads were conducted on the Biodex Medical System 3^®^ dynamometer (Biodex Medical Systems, Shirley, New York), in accordance with the procedures of previous studies [16, 20, 21]. Measurements were performed unilaterally on the right side, unless there was a medical contraindication. Participants were seated on a backward-inclined (5°) chair, which is part of the dynamometer. A strap was applied across the thigh on the test side and the hips and shoulders were stabilized with safety belts to avoid additional movements. The rotational axis of the dynamometer was aligned with the transversal knee-joint axis and connected to the point of force application at the distal end of the tibia (i.e. 5cm above the lateral malleolus) using a length-adjustable rigid lever arm. Range of motion was set from a knee joint angle of 90° to 160°, with a fully extended leg corresponding to a knee angle of 180°. The test protocol included two standardized tests in the following order: isometric and isotonic tests. During each test, the subjects were clearly instructed by the test leader to perform the tests with maximal effort. The protocol was conducted twice per test session.

*Isometric test*. Isometric strength of the knee extensors was assessed at a knee joint angle of 90°. Subjects were instructed to avoid an explosive contraction, but to extend their leg as hard as possible during 5 seconds, by building up strength gradually until maximal strength was reached. Two maximal isometric knee extensions were performed separated by a 10-second rest interval. Peak torque (pT, Nm) was recorded.

*Isotonic tests*. The isotonic tests included ballistic knee extensions against constant resistances of consecutively 50%, 25%, 0% and 75% of the maximum isometric strength in a knee joint angle of 90°. Because of practical restrictions, the test at 0% of the isometric maximum was conducted at a fixed resistance of 1Nm, which can be considered as an unloaded test. The subjects were asked to extend their leg as fast and as hard as possible and then passively return the leg to the starting position (90°). Two explosive contractions were performed. Velocity (°/s) and torque (Nm) were recorded.

#### Signal processing

Torque and velocity signals were sampled at 100 Hz and processed off-line using a commercial software package (Matlab R2015b, The MathWorks Inc., Natick, Massachusetts, United States). Instantaneous power (Nm/s) was calculated as the product of torque (Nm) and velocity (rad/s). Peak velocity (pV, °/s) and peak power (pP, Nm/s) were determined as the highest values of the velocity curve and power curve respectively. Rate of velocity development (RVD, °/s^2^) and rate of power development (RPD, Nm/s^2^) were calculated as the linear slopes of the velocity- and power-time curve respectively, from the start of the movement until pV or pP was reached (RVD and RPD) as well as at time intervals of 50ms (*RVD*_0–50_ or *RPD*_0–50_, *RVD*_50–100_ or *RPD*_50–100_ and *RVD*_100–150_ or *RPD*_100–150_). The start of the movement was determined as the point where the acceleration reached a threshold of 150 °/s^2^ after overcoming the imposed load. The tests with the highest pT for the isometric tests and the highest pV for the isotonic tests were used for further analyses.

#### Statistical analyses

All analyses were performed using R software version 3.2.2. Statistical significance was set at p < 0.05. All data were screened for normality using the Shapiro Wilk test. To confirm the absence of systematic bias between test and retest, paired t-tests were calculated [28].

Relative reliability, the degree to which the subjects maintain their position in a sample over the repeated measures, was assessed using intraclass correlation coefficients (*ICC*_3,1_) and their 95% confidence intervals (95% CI). Computation of ICC was based on repeated measures analysis of variance using a single rating, two-way mixed effects model where subject effects are random and rater is a fixed effect [29, 30]. As no universally acceptable levels have been adopted for correlation coefficients in describing the amount of reliability, we used previously reported ICC ranges of Youdas et al. [31]: >.90 high, .89–.80 good, .79–.70 fair, < .69 poor reliability.

In addition, absolute reliability, the degree to which the repeated measures vary for the subjects, was expressed in terms of standard error of measurement (SEM) and limits of agreement (LoA) [32]. SEM was determined as the square root of the residual mean square error from the analysis of variance [33, 34]. In addition, the reference interval of the difference scores between test and retest data, defined as the limits of agreement (LoA), was calculated as the mean difference ± 1.96 x the standard deviation from the differences between test and retest [32]. Both SEM and LoA were expressed as a percentage of the mean to make interpretation and comparison among different measures and across different studies possible. SEM was also expressed in the unit of the measured variable. SEM represents 68% of the error between test and retest for the average individual in the sample, whereas the more strict LoA represent the test-retest differences for 95% of the whole population [32].

## Results

There was no drop out. However, 28% of the subjects were not able to properly overcome the resistance for the isotonic tests at 75% load, resulting in the systematic failure of the test and the retest. Consequently, isotonic testing at 75% can be considered as systematically unreliable and was excluded for further analyses. Therefore, we will focus on the isotonic tests at 0%, 25% and 50% load.

The Shapiro Wilk test allowed us to assume normality for all parameters at a significance level of 0.01. Mean ± standard deviation (SD) of isometric peak Torque (pT) was 144.9 ± 45.9 Nm for the test and 143.6 ± 44.3 Nm for the retest session. Test and retest data of the isotonic tests are represented in Table 1. Paired t-tests indicated that no data were significantly different between test and retest (p > 0.05).

**Table 1:** Test and retest data (N = 63 (♂ 27, ♀ 36)).

Parameters	0%				25%				50%			
	Test		Retest		Test		Retest		Test		Retest	
	Mean	SD	Mean	SD	Mean	SD	Mean	SD	Mean	SD	Mean	SD
***pV* (°/*s*)**	402.3	51.4	401.4	55.2	292.0	44.2	290.4	40.5	169.5	41.7	169.7	38.1
***pP* (*Nm*/*s*)**	244.0	90.0	242.1	91.2	293.3	118.2	298.0	119.5	266.6	118.4	270.7	115.3
***RVD* (°/*s*^2^)**	1735.3	495.4	1720.1	512.6	1100.4	296.4	1093.2	270.5	681.4	187.7	688.9	179.0
***RVD***_**0–50**_ **(°/*s*^2^)**	1217.5	756.5	1115.2	754.2	870.9	456.7	854.9	384.7	522.7	223.9	521.0	226.2
***RVD***_**50–100**_ **(°/*s*^2^)**	2013.0	818.2	1997.3	834.6	1381.1	530.6	1379.8	462.4	924.2	295.3	942.5	295.2
***RVD***_**100–150**_ **(°/*s*^2^)**	2048.7	570.9	2096.1	583.4	1493.8	402.2	1518.2	368.3	973.2	287.2	983.9	271.4
***RPD* (*Nm*/*s*^2^)**	1318.1	648.2	1320.4	725.4	1360.9	702.1	1379.0	694.7	1215.9	629.8	1251.9	623.7
***RPD***_**0–50**_ **(*Nm*/*s*^2^)**	885.4	802.7	814.6	745.3	1030.2	765.9	996.8	666.2	931.5	658.1	942.6	617.2
***RPD***_**50–100**_ **(*Nm*/*s*^2^)**	1617.7	958.2	1641.5	1025.3	1767.8	1092.4	1793.1	1083.1	1750.4	1012.7	1806.9	971.9
***RPD***_**100–150**_ **(*Nm*/*s*^2^)**	1340.6	833.9	1448.2	792.1	1704.0	847.5	1817.8	809.2	1601.2	791.6	1642.7	807.5

Mean and standard deviation (SD) of the test and retest session for peak velocity (pV), peak power (pP), rate of velocity development until pV (RVD) and at intervals of 50ms (***RVD***_**0–50**_, ***RVD***_**50–100**_, ***RVD***_**100–150**_) and rate of power development until pP (RPD) and at intervals of 50ms (***RPD***_**0–50**_, ***RPD***_**50–100**_, ***RPD***_**100–150**_) measured during the isotonic tests at 0%, 25% and 50% of maximal isometric strength. No test and retest sessions were significantly different from each other at p < 0.05.

Isometric pT showed very high relative reliability with an ICC of .97. For the isotonic tests, ICCs were good to high for pV, pP, RVD and RPD at all resistances (Table 2). *RVD*_0–50_ showed poor reliability at 0% and 50% load and fair reliability at 25% load. At time intervals of 50-100ms and 100-150ms, RVD showed fair to good reliability and RPD good to high reliability across all loads.

**Table 2:** Relative and absolute reliability (N = 63 (♂ 27, ♀ 36)).

Parameters	0%				25%				50%			
	ICC (95% CI)	SEM	SEM (%)	LoA (%)	ICC (95% CI)	SEM	SEM (%)	LoA (%)	ICC (95% CI)	SEM	SEM (%)	LoA (%)
***pV* (°/*s*)**	.96 (.93-.97)	11.3	3	0 ± 9	.90 (.84-.94)	13.5	5	0 ± 14	.85 (.76-.91)	15.5	9	-2 ± 29
***pP* (*Nm*/*s*)**	.97 (.95-.98)	16.4	7	1 ± 20	.98 (.97-.99)	16.4	6	-2 ± 20	.96 (.94-.98)	23.3	9	-4 ± 37
***RVD* (°/*s*^2^)**	.95 (.91-.97)	117.0	7	1 ± 22	.92 (.87-.95)	80.8	7	-1 ± 24	.89 (.82-.93)	61.1	9	-3 ± 30
***RVD***_**0–50**_ **(°/*s*^2^)**	.66 (.50-.78)	438.9	38	-12 ± 174	.78 (.66-.86)	198.6	23	-9 ± 90	.62 (.43-.75)	139.6	27	-10 ± 93
***RVD***_**50–100**_ **(°/*s*^2^)**	.83 (.74-.90)	336.9	17	-10 ± 118	.83 (.74-.90)	203.5	15	-6 ± 64	.74 (.60-.83)	151.4	16	-7 ± 91
***RVD***_**100–150**_ **(°/*s*^2^)**	.86 (.78-.91)	214.4	10	-5 ± 44	.87 (.80-.92)	137.9	9	-4 ± 38	.85 (.77-.91)	106.5	11	-3 ± 37
***RPD* (*Nm*/*s*^2^)**	.97 (.95-.98)	125.9	10	1 ± 34	.97 (.95-.98)	126.2	9	-4 ± 33	.96 (.94-.98)	120.9	10	-6 ± 43
***RPD***_**0–50**_ **(*Nm*/*s*^2^)**	.76 (.63-.85)	381.3	45	-47 ± 354	.88 (.80-.92)	251.8	25	-17 ± 119	.83 (.74-.90)	260.0	28	-11 ± 85
***RPD***_**50–100**_ **(*Nm*/*s*^2^)**	.85 (.77-.91)	379.7	23	-18 ± 159	.94 (.90-.96)	271.8	15	-11 ± 94	.92 (.88-.95)	272.6	15	-9 ± 76
***RPD***_**100–150**_ **(*Nm*/*s*^2^)**	.81 (.71-.88)	350.7	25	-21 ± 183	.92 (.87-.95)	236.1	13	-12 ± 53	.91 (.86-.95)	236.1	15	-7 ± 53

Relative and absolute reliability for peak velocity (pV), peak power (pP), rate of velocity development until pV (RVD) and at intervals of 50ms (***RVD***_**0–50**_, ***RVD***_**50–100**_, ***RVD***_**100–150**_) and rate of power development until pP (RPD) and at intervals of 50ms (***RPD***_**0–50**_, ***RPD***_**50–100**_, ***RPD***_**100–150**_) measured during the isotonic knee extension tests at 0%, 25% and 50% of maximal isometric strength: intraclass correlation coëfficients (ICC) with 95% confidence intervals (CI), standard error of measurement (SEM) and limits of agreement (LoA). All ICCs were significant at p < .001.

**Fig 1:**
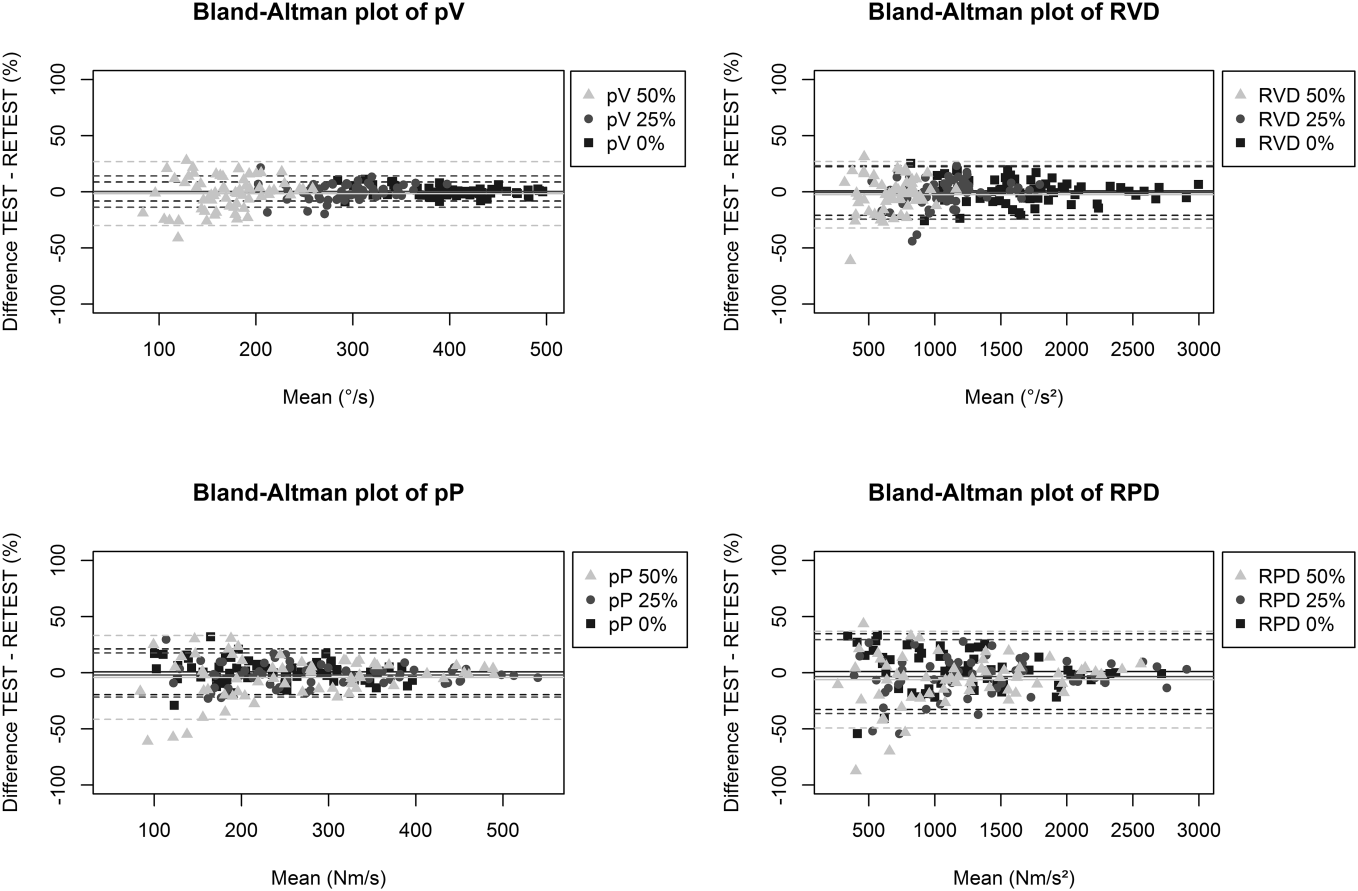
Bland-Altman plots. Bland-Altman plots of peak velocity (pV), rate of velocity development (RVD), peak power (pP) and rate of power development (RPD) illustrate absolute reliability of the isotonic knee extension tests at 0%, 25% and 50% of isometric maximum strength. Individual differences between test and retest values are plotted against their means. For all plots at all loads the mid solid line represents the mean difference, whereas the outer dashed lines represent the limits of agreement (LoA) in percentage.

Absolute reliability for isometric pT was represented by SEM (5.7%) and LoA (-1.1% ± 16.8%). Absolute reliability indices of RVD and RPD (i.e. SEM 7–10%) were good and similar to indices of pV and pP (i.e. SEM 3–9%) across all loads (Table 2). Early phase measures of acceleration capacity (i.e., the first 50 ms) showed poor absolute reliability (i.e. SEM 23–45%). RVD and RPD showed good to fair absolute reliability (i.e. SEM 9–25%) at time intervals of 50-100ms and 100-150ms. LoA of the parameters obtained in the isotonic tests at lower load tended to be smaller compared to the tests at higher load, as visualised using the Bland-Altman plots (Fig 1). Bland-Altman plots show that LoA were situated symmetrically around the zero line and that variability decreased with higher test scores [35]. In other words, no systematic error in the outcome measures was observed and reliability increased with increasing performance capacities.

## Discussion

The aim of this study was to evaluate reliability of time-dependent measures of knee extensor muscle function using the isotonic mode on a Biodex System 3 dynamometer. The present results indicate high test-retest reliability for all isotonic measurements at relatively low loads (i.e. 0%, 25% and 50%) in an older population. Similar to frequently reported parameters like pV and pP, RVD and RPD were found to be reliable. Importantly, reliability of muscle function seemed to decrease with increasing load and with decreasing test performance. The acceleration phase of the isotonic leg extension movements (i.e. first 50ms) was highly sensitive to variation and should be interpreted with caution because of poor reliability. Overall, this study demonstrates that several reliable parameters can be identified to provide a basis for a more precise and sensitive use of the isotonic tests in clinical practice.

Loaded isotonic tests were performed relative to maximal strength. Therefore, reliability of these tests was partly based on reliability of the maximum strength tests. The present study showed very high reliability for the isometric test. This finding is in line with former research (ICC: .87-.98), which used isokinetic dynamometry to measure isometric knee extension strength in a knee angle of 90° in elderly using a similar procedure as in the present study [36, 37]. In addition, the mean difference between test and retest data was about 1% and the SEM was 6%. Given that strength training interventions in older adults have been found to induce gains of approximately 10% to 20% in isometric muscle strength [38], the present results indicate that the isometric tests on an isokinetic dynamometer are capable of assessing real training-induced changes.

Isotonic pV and pP are regularly reported in the literature. On the one hand, pV gives an indication of a subject’s pure velocity capacities independent of the strength capacities. On the other hand, taking into account torque and velocity by calculating power gives a better indication of the subject’s overall muscle capacities. This study demonstrated high relative reliability at relatively low loads (i.e. 0%, 25% and 50%) for pV and pP (ICC: .85–.98). Mean differences between test and retest data for pV ranged from -2% to 0% and for pP from -4% to 1%. Previous research has investigated the reliability of ankle plantar- and/or dorsiflexor tests at low loads (1Nm to 50% load) using the isotonic mode of an isokinetic dynamometer. The ICC’s of pV and pP at 20% load reported by Power et al. [15] in young men and women, i.e. .93 and .98 respectively, are similar to the ICC’s for these parameters in our study. Likewise, ICC’s reported by Webber et al. [33] were fair to high for pV and pP in older women, ranging from .76 to .96, with mean differences ranging from -2% to 2% for pV and from -6% to -1% for pP.

Across the different loads used in this study, variability between test and retest for all parameters tended to decrease with increasing mean test performance (Fig 1). In other words, the better the performance of the subject the more reliable the measurement. In addition, absolute reliability of pV and RVD tended to be lower with higher loads compared to lower loads (SEM: 3%–9%). The same trend of increased variability with increasing loads was illustrated on Bland and Altman plots (LoA: 9%–30%) (Fig 1). As opposed to testing at relatively low loads, isotonic testing at high load (i.e. 75%) should be avoided, because of the total failure to perform these tests properly for 28% of the sample subjects. The failure of the isotonic tests at high load might have been related to the greater challenge for the subjects to generate sufficient torque throughout the entire range of motion [7]. While maximal dynamic strength testing on weight stack machines at high loads could be used for comparing absolute strength improvements due to training (i.e. 1-RM testing), lower loads should be advised for isotonic testing on an isokinetic dynamometer to be able to reach full range of motion. Power et al. [15] indicated that a load of 20% of the isometric maximum represents a moderate resistance in which all subjects can perform fast, explosive contractions without range of motion failure. In line with this statement, the results of the current study indicate that relatively low loads (i.e. 25% and 50%) are feasible and highly reliable. In addition, they support the use of isotonic tests at 0% load. Although many daily activities include a minimal load to be moved, it has previously been shown that peak velocity of an unloaded movement (i.e. 0% load) is a key component in the onset of functional difficulties in the elderly [16].

The main instruction to the subjects when conducting rapid strength tests is ‘push as fast and as hard as possible’ [39]. Consequently, a certain challenge of performing explosive tests is to reach pP as fast as possible. However, pV and pP do not provide information on the development of velocity or power throughout the knee extension movement. RVD and RPD take into account the time needed to develop velocity or power, respectively. Moreover, RVD and RPD are shown to differentiate more between young and older individuals, which emphasizes their potential value in aging research [24, 25]. Therefore, reliability studies on these parameters are crucial. To our knowledge, this is the first study that evaluated reliability indices of RVD and RPD of the knee extensor muscles obtained through isotonic testing. Previously, Webber et al. [33] calculated the average acceleration obtained from the isotonic tests of the ankle plantar- and dorsiflexors at ~0% and 50% load and reported fair to high reliability. Similarly, our results show that the ICCs of RVD and RPD were high for the isotonic tests at 0%, 25% and 50% load (ICC: .89–.97). Therefore, RVD and RPD can be used in future research to reliably evaluate age-related changes in muscle function or adaptations after an intervention.

To gain more insight into the development of an explosive knee extension movement, different time phases of the acceleration phase were examined. In line with previous approaches during isometric testing [40, 41], RVD and RPD were calculated over different time windows of 50ms each. The early phase of explosive contractions (0-50ms), which has been suggested to be predominantly determined by neural activation characteristics [42, 43], demonstrated poor absolute reliability for the isotonic tests in this study (SEM: 23%–45%). Next to the starting phase, parameters calculated at time frames of 50-100ms and 100-150ms, which have been suggested to be predominantly determined by muscular characteristics [42, 43], demonstrated fair to high reliability (ICC: .74–.94, SEM: 9%–25%). In agreement with our results, Buckthorpe et al. [44] found that the most reliable rate of force development window, measured during an isometric contraction, lies between 50 and 100ms. They found that the first 50ms, the early phase of rapid force development, demonstrated poor absolute reliability (CV: 16.6–18.7%), which was significantly worse than the reliability indices for 0-100ms (CV: 6.4–9.8%) and for 0-150ms (CV: 5.1–8.4%). Accordingly, Buckthorpe et al. [44] suggested that the variability of the early phase of an explosive contraction is likely due to an inherent variability in neural drive.

An important strength of our study is the use of isotonic tests to evaluate the reliability of RVD and RPD. Although less used, isotonic tests are considered to be more functionally relevant in older adults as compared to isometric or isokinetic tests, because they include dynamic movements at fixed loads with an unconstrained velocity which replicates most daily activities. In addition, isotonic tests include rapid power development suggesting a different physiological basis than maximal muscle strength testing [17]. Even more important, the lower incidence of a Valsalva maneuver and consequently the smaller acute elevation in blood pressure [45] may have resulted in the participants’ experience that the isotonic tests were a lot more comfortable and safer to perform, which is especially relevant in the older population. In addition, there is an increased interest in using fast velocity and power measurements to identify age-related differences in muscle function [24]. However, the following limitations of the study should be taken into account. First, isotonic testing at 75% load led to range of motion failure for 28% of the sample. Therefore, only three loads were analyzed. Second, no conclusions can be made about the level of load that would result in poor reliability outcomes, because no continuum was measured. Third, the 95% LoA presented in this study may be too strict as decision limits for clinical practice and may have been more valuable for practice if tested with a larger sample. The larger the sample, the better the mean and standard deviation of the residuals will approach the real values of the distribution. Nevertheless, in comparison with most reliability studies, our sample size was high. To see a true change in performance for a group of older individuals, the degree of certainty of 95% LoA may be unrealistic and we therefore recommend looking at the SEM for practical applications. The SEM can be interpreted as the limits for change required to indicate a real increase or decrease for a group of individuals following an intervention [33]. Though, calculation of the LoA is of practical importance as it provides the minimal change for an individual needed to be considered real [30, 46].

In conclusion, this study demonstrated that knee extension velocity and power (i.e. pV and pP) are reproducible at relatively low loads (i.e. 0%, 25% and 50%). At 75% load, isotonic testing was found to be unreliable. The use of loads ≥ 75% of maximal isometric strength in 90° knee angle should be avoided for isolated isotonic knee-extension tests on isokinetic dynamometers, because this load seems too high to allow for an adequate test over the full range of motion. In addition, this study indicated that time-dependent measures of muscle function (i.e. RVD and RPD), which seem to be more sensitive to detect early age-related changes [25], are equally reliable compared to maximal measures (i.e. pV and pP). However, caution is advised when interpreting early phase results. Furthermore, a trend for increased reliability with decreasing load and with increasing mean test performance was shown. These results indicate that isotonic testing on an isokinetic dynamometer can be used as a consistent measure of rapid velocity and power capacities for a group of older individuals, in repeated measures designed studies. This is of high clinical relevance, given that muscle power development and movement velocity are emerging as the primary outcomes for preventive and rehabilitative interventions in aging research, due to their predictive value for functional impairments and disability.
